# A Combined Molecular Cloning and Mass Spectrometric Method to Identify, Characterize, and Design Frenatin Peptides from the Skin Secretion of *Litoria infrafrenata*

**DOI:** 10.3390/molecules21111429

**Published:** 2016-10-26

**Authors:** Di Wu, Yitian Gao, Lei Wang, Xinping Xi, Yue Wu, Mei Zhou, Yingqi Zhang, Chengbang Ma, Tianbao Chen, Chris Shaw

**Affiliations:** 1 Natural Drug Discovery Group, School of Pharmacy, Queen’s University, Belfast BT9 7BL, Northern Ireland, UK; dwu03@qub.ac.uk (D.W.); ygao07@qub.ac.uk (Y.G.); l.wang@qub.ac.uk (L.W.); x.xi@qub.ac.uk (X.X.); ywu16@qub.ac.uk (Y.W.); m.zhou@qub.ac.uk (M.Z.); t.chen@qub.ac.uk (T.C.); chris.shaw@qub.ac.uk (C.S.); 2 Department of Emergency Medicine, The First Hospital of Hebei Medical University, Shijiazhuang 050031, China

**Keywords:** frog skin secretion, antimicrobial peptide, frenatin, modification, structure activity relationship

## Abstract

Amphibian skin secretions are unique sources of bioactive molecules, particularly bioactive peptides. In this study, the skin secretion of the white-lipped tree frog (*Litoria infrafrenata*) was obtained to identify peptides with putative therapeutic potential. By utilizing skin secretion-derived mRNA, a cDNA library was constructed, a frenatin gene was cloned and its encoded peptides were deduced and confirmed using RP-HPLC, MALDI-TOF and MS/MS. The deduced peptides were identified as frenatin 4.1 (GFLEKLKTGAKDFASAFVNSIKGT) and a post-translationally modified peptide, frenatin 4.2 (GFLEKLKTGAKDFASAFVNSIK.NH_2_). Antimicrobial activity of the peptides was assessed by determining their minimal inhibitory concentrations (MICs) using standard model microorganisms. Through studying structure–activity relationships, analogues of the two peptides were designed, resulting in synthesis of frenatin 4.1a (GFLEKLKKGAKDFASALVNSIKGT) and frenatin 4.2a (GFLLKLKLGAKLFASAFVNSIK.NH_2_). Both analogues exhibited improved antimicrobial activities, especially frenatin 4.2a, which displayed significant enhancement of broad spectrum antimicrobial efficiency. The peptide modifications applied in this study, may provide new ideas for the generation of leads for the design of antimicrobial peptides with therapeutic applications.

## 1. Introduction

Although peptide drugs, such as insulin, were discovered in the last century, small molecules are still preferred in the drug development process, mainly because of their ease of production, simpler administration routes and superior pharmacodynamic properties. However, in the 21st century, the modern drug industry has gradually advanced to a new frontline. Protein- and polypeptide-based drugs have been seriously taken into account because of their multifarious structures and high affinity for many targets, as well as the fact that these agents have provided natural lead compounds for drug development [1].

Amphibians, like frogs and toads, have evolved various defence mechanisms for survival. Their bioactive peptides, especially the antimicrobial peptides from skin secretions, have attracted much attention during the past decades. To date, 1007 active amphibian peptides have been included in the Antimicrobial Peptides Database [2]. The disruption and permeabilization of microbial cell membranes are the most effective ways to prevent microbes from developing resistance because the composition of microbial membranes is evolutionarily stable, making it less likely to acquire resistance through mutation [3]. This important natural property of antimicrobial peptides represents a fundamental quality for therapeutic potential.

In this study, the previously characterized peptide, frenatin 4.1 and the novel post-translationally modified peptide, frenatin 4.2, were isolated from the skin secretion of *Litoria infrafrenata*. The structures of the peptides were obtained via “shotgun” cloning using 3′-Rapid amplification of cDNA ends (3′-RACE) and characterized by MS/MS sequencing. The synthetic replicates of the peptides were subjected to antimicrobial and haemolysis assays to evaluate their bioactivities and toxicities, respectively. Since the antimicrobial activities were not as good as expected, amino acid substitutions were made to obtain analogues with enhanced efficiency. In light of data derived from structure-activity relationships, analogues with more potent antimicrobial activities were designed, chemically synthesized and their bioactivities were evaluated.

## 2. Results

### 2.1. “Shotgun” Cloning of Peptide Precursor-Encoding cDNAs

**Figure 1:**
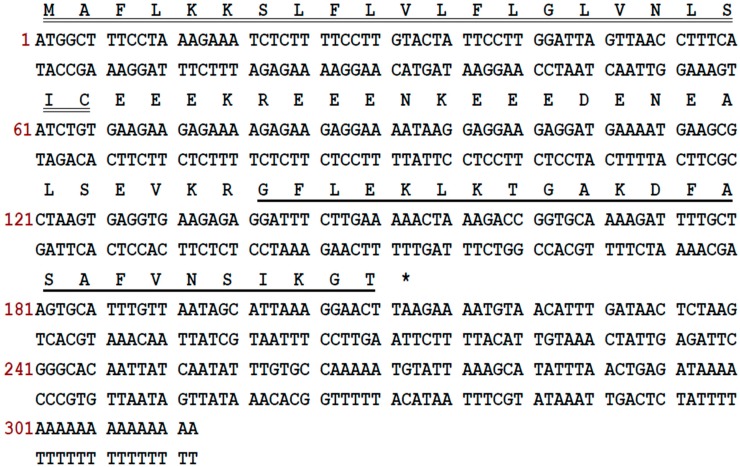
Nucleotide sequence of the cDNA cloned from *L. infrafrenata* skin secretion and its predicted peptide sequence. The putative signal peptide (double-underlined), mature peptide (single-underlined) and stop codon (asterisk) are indicated.

Through 3′-RACE, a frenatin peptide precursor-encoding cDNA was cloned from the skin secretion cDNA library of *L. infrafrenata* (Figure 1). The encoded peptide precursor consisted of 70 amino acid residues. It comprised a putative signal peptide of 22 residues terminating with a cysteine, a sequence of a mature peptide of 24 amino acid residues and an acidic spacer peptide between these two regions. This peptide precursor had been identified previously and the mature peptide was named frenatin 4.1 [4].

### 2.2. Structural Characterisation of Frenatins from Reversed Phase HPLC Fractions of Skin Secretion

**Figure 2:**
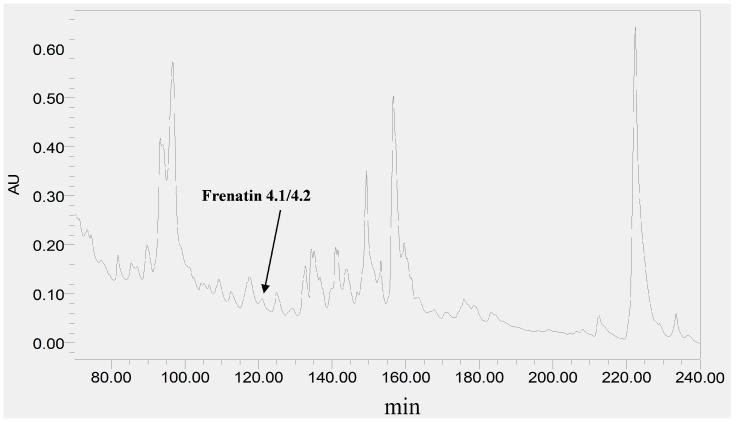
Region of RP-HPLC chromatogram of *L. infrafrenata* skin secretion with an arrow indicating the elution position/retention time of both frenatin 4.1 and its natural post-translationally modified analogue, frenatin 4.2.

**Figure 3:**
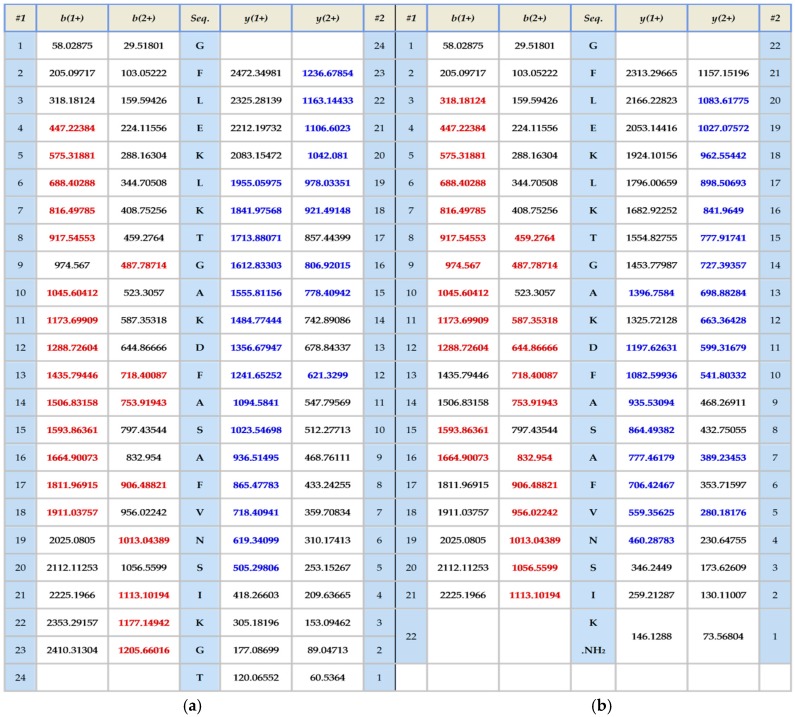
MS/MS fragmentation datasets of fragment ions corresponding to those of frenatin 4.1 (**a**) and frenatin 4.2 (**b**). Expected singly- and doubly-charged b-ion and y-ion fragment *m*/*z* ratios were predicted online using Protein Prospector [5]. Observed fragment ions are indicated in red- and blue-coloured typefaces.

The skin secretion was fractionated by RP-HPLC and the column effluent was monitored by UV absorbance at 214 nm and 280 nm. Figure 2 shows the relevant region of the RP-HPLC chromatogram at 214 nm. The eluted fractions were collected at one minute intervals and analyzed by MALDI-TOF MS. The masses of the peptides in fractions were recorded and compared with the theoretical peptide masses deduced from the cDNA. A peptide with a molecular mass identical to frenatin 4.1 was found in reverse phase HPLC fraction #122 and another peptide of *m*/*z* 2371.21 of lower abundance, was also present and was deemed to be a post-translationally modified version of frenatin 4.1. The primary structures of each peptide were unambiguously determined by MS/MS fragmentation sequencing and the post-translationally modified peptide, which was named frenatin 4.2, was also confirmed (Figure 3).

Both frenatins and their analogues were successfully synthesized by solid-phase 9-Fluorenylmethyloxycarbonyl (Fmoc) chemistry and purified by reversed phase HPLC. Their sequences and degrees of purity were established by MALDI-TOF mass spectrometry and analyzed by MS/MS fragmentation.

### 2.3. Secondary Structures and Physicochemical Properties of the Frenatins

**Figure 4:**
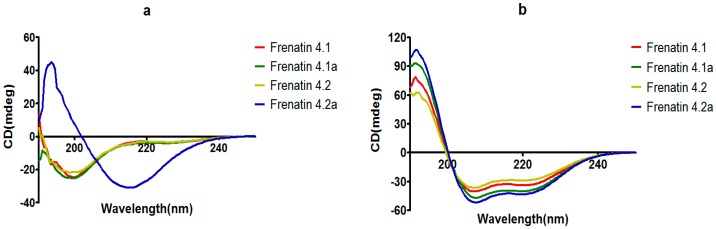
CD spectra recorded for frenatins and their analogues (100 μM) in (**a**) in 10 mM ammonium acetate water solution and (**b**) in 50% 2,2,2-trifluoroethanol (TFE)/10 mM ammonium acetate water solution.

**Figure 5:**
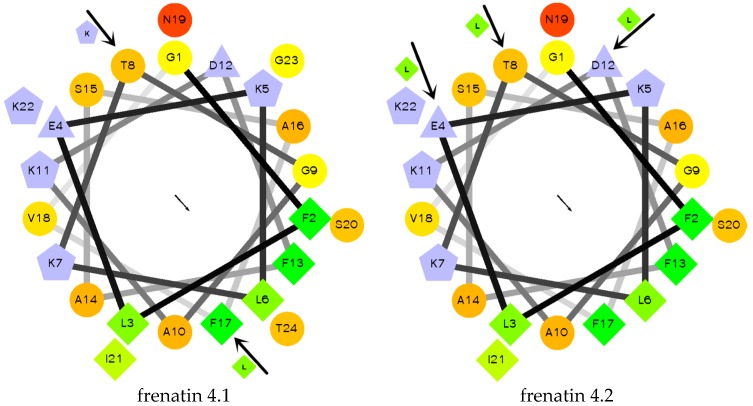
Helical wheel projections of frenatin 4.1, frenatin 4.2 and amino acid residue substitutions of their analogues. The directions of hydrophobic moments of the parent peptides are denoted by the arrows in the middle of the wheels. The amino acid residue substitutions are also indicated.

The secondary structures of the peptides were determined by circular dichroism (CD) in 10 mM ammonium acetate/water solution and 50% 2,2,2-trifluoroethanol (TFE) in 10 mM ammonium acetate water solution, respectively. Except for frenatin 4.2, which adopted a β-sheet conformation, the other three peptides existed in random coil form in ammonium acetate solution (Figure 4a). However, in the membrane mimetic medium (TFE solution), all of the peptides were induced to form typical α-helical structures (Figure 4b). The CD data (190 nm to 240 nm, 1 nm pitch) of the peptides in TFE solution was submitted to the K2D3 web server to estimate the α-helical content. The results showed that the helicities of the modified analogues increased in expectation (Table 1). Helical wheel projections of both frenatin 4.1 and frenatin 4.2 were plotted in Figure 5. These plots illustrated, to a certain extent, the amphipathic feature of both peptides. The two analogues frenatin 4.1a and frenatin 4.2a were designed by several amino acid substitutions (indicated in Figure 5) based on the structure–activity relationship. The major physicochemical properties of the four peptides are summarised in Table 1.

### 2.4. Antimicrobial/Haemolytic Activities of the Peptides

Antimicrobial activity of the peptides was assessed through establishing their minimal inhibitory concentrations (MICs) using standard model microorganisms: the Gram-positive bacterium, *Staphylococcus aureus* (NCTC 10788), the Gram-negative bacterium, *Escherichia coli* (NCTC 10418) and the opportunistic yeast pathogen, *Candida albicans* (NCPF 1467). The MICs obtained are shown in Table 2.

Overall, the naturally-occurring peptide, frenatin 4.1, and the post-translationally modified peptide, frenatin 4.2, were found to be ineffective against the Gram-positive bacterium, *S. aureus,* at concentrations up to 512 μg/mL. Moreover, frenatin 4.1 also showed no activity against the Gram-negative bacterium, *E. coli* and the potentially-pathogenic yeast, *C. albicans,* at concentrations up to 512 μg/mL, while frenatin 4.2 exhibited a weak activity against *E. coli* and *C. albicans*. Compared with frenatin 4.1, its analogue, frenatin 4.1a, exhibited more potent activity against *E. coli* and *C. albicans* but was still ineffective against *S. aureus*. However, the analogue of frenatin 4.2, frenatin 4.2a, showed greatly improved activities against the growth of all three test microorganisms. The effects of amino acid substitutions were reflected in a four-fold increase in inhibition potency against *E. coli*, a 16-fold increase against *C. albicans*, and, more importantly, an inhibition against *S. aureus* emerged, making this synthetic analogue a broad-spectrum antimicrobial peptide.

**Figure 6:**
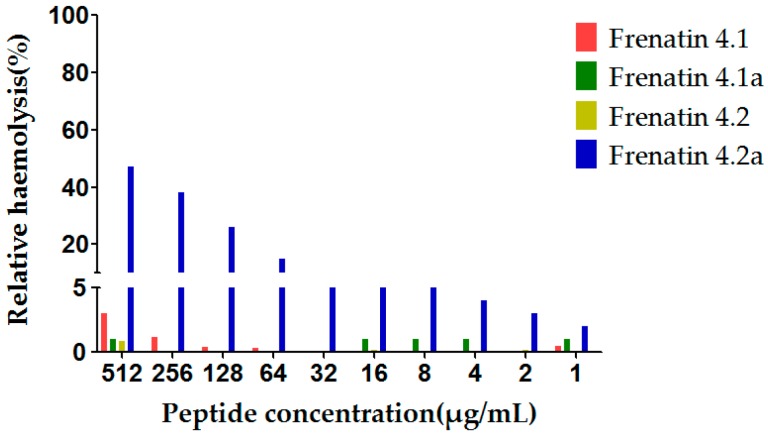
Relative haemolysis of the frenatin 4.1, 4.2 and their analogues. The 100% haemolysis was defined using haemolytic effect induced by 1% TritonX-100 (Sigma-Aldrich, St. Louis, MO, USA).

The potent modified peptide analogue, frenatin 4.2a, demonstrated detectable haemolytic activity at the highest concentration tested, while displaying little activity at the lower range of concentrations tested, 16 μg/mL or 32 μg/mL, of which latter concentrations corresponded to the MICs against the test microorganisms. None of the other three peptides displayed observable haemolytic activity in the tested concentration range, which correlated with their weak antimicrobial activities (Figure 6).

## 3. Discussion

Increasing numbers of researchers are focusing on peptides because they possess good functional activities and few side effects, as well as providing natural lead compounds for the development of a variety of drugs. The habitats of amphibians are usually also beneficial to microorganisms, which makes amphibians possibly susceptible to infections. However, amphibians, such as frogs and toads, have evolved various defence mechanisms to protect themselves against this eventuality. Their skin secretions contain a large number of bioactive molecules and some of these are antimicrobial peptides that play an important role in their defence against pathogenic microbes. Their membrane disrupting and permeabilizing mechanisms also make it difficult for microbes to acquire relevant resistance to these peptides through mutation.

*Litoria infrafrenata* is known as the white-lipped tree frog or giant tree frog and it belongs to the Hylidae family. The habitat of *L. infrafrenata* is located along the coastal areas of the Cape York Peninsula in Queensland, Australia, but is also widely-distributed in the New Guinea region. Thus far, only a few frenatins have been discovered (Table 3). Some of these sequences were determined by a combination of automated Edman sequencing and mass spectrometry. Furthermore, some frenatins such as frenatin 1.1, frenatin 3.1 as well as frenatin 4.1 and frenatin 4.2 (described in this study), were discovered through molecular cloning technology, which has made it possible to obtain information on their precursors.

In the case of frenatin 4.1, the precursor is composed of a signal peptide followed by an acidic spacer peptide which is flanked by typical amino acid lysine-arginine (K-R), and a mature peptide. The putative 22-amino acid residue signal peptide is highly-conserved. In fact, antimicrobial peptides from the frogs of the Hylidae and Ranidae families appear to originate from a common 150-million-year-old ancestral precursor as shown by the highly-conserved signal peptide, although the mature peptides contain much sequence variation [9]. The peptide precursor can be delivered to the gland by the signal peptide, and, from there, the signal peptide is removed by endoprotease. The remainder of the peptide still remains inactive until an external stimulation is received. The acidic spacer peptide is also cleaved, and the mature peptide is finally secreted onto the skin surface. The acidic spacer peptide, located between the mature peptide and signal peptide, is also highly-conserved among most antimicrobial peptides, but its function has not been clearly established [10]. Using RP-HPLC, MALDI-TOF and MS/MS, we have successfully identified the post-translationally modified form of frenatin 4.1 as a C-terminally amidated peptide (frenatin 4.2). The glycine residue at the second position from the C-terminus of frenatin 4.1 was found to act as the amide donor during the amidation reaction.

The antimicrobial activities of frenatin 4.1 and frenatin 4.2 have not been reported previously. Therefore, we assessed the antimicrobial activity of both peptides against three different species of microorganisms. Unfortunately, the expected antimicrobial activities of the two peptides were not observed, so attempts were made to modify the parent peptides by substituting selected amino acid residues. In structure-activity relationship studies of antimicrobial peptides, there are four main factors that contribute to antimicrobial activity. These are α-helicity, cationic charge, hydrophobicity and hydrophobic moment, but the antimicrobial activity and cell selectivity are affected by a dependent combination of a variety of parameters, which made it hard to estimate the contributions of the physicochemical parameters. Firstly, considering Thr^8^ is short of cationicity and the large aromatic side chain of Phe^17^ may destabilize α-helices in frenatin 4.1., the analogue frenatin 4.1a was designed by substituting Thr^8^ and Phe^17^ in frenatin 4.1, by Lys and Leu, respectively, to increase the net charge (from +2 to +3), hydrophobic moment (from 0.491 to 0.540) and helicity (from 43.87% to 55.41%) of the peptide. Antimicrobial activity of this peptide, frenatin 4.1a, against *E. coli* and *C. albicans*, was then observed. Subsequent re-evaluation of the physicochemical properties of frenatin 4.1a, showed that its hydrophobicity was still relatively low (0.244), even lower than that of frenatin 4.1 (0.300). The post-translationally modified peptide, frenatin 4.2, showed stronger activities against *E. coli* and *C. albicans* compared to frenatin 4.1. Frenatin 4.2 is a C-terminally amidated peptide and thus has one more positive charge than frenatin 4.1, which has a free acid C-terminus, and through secondary structure prediction, the C-terminal Gly-Thr of frenatin 4.1 adopts a random coil conformation that does not contribute to its helicity. The improvement of the antimicrobial activities of frenatin 4.2 may be contributed by its higher positive charge, which enables the peptide to reach an effective concentration on the negatively-charged bacterial membrane. It is reported that the C-terminal amidation of antimicrobial peptides has a flexible effect on their antimicrobial activity and no obvious effect on their selectivity [11]. However, amidation of the C-terminal of a peptide can eliminate or reduce its proteolytic degradation, effectively enhancing its stability [12]. Thus, frenatin 4.2 became the focus for further modifications.

Hydrophobicity of an antimicrobial peptide must be kept within a certain range because low hydrophobicity would reduce the membrane insertion ability of the peptide, resulting in decreased antimicrobial activity [13]. Cationic charge also contributes to antimicrobial activity as aforementioned. However, the cationic charge of a peptide and its antimicrobial activity are also not absolutely positively-correlated. For example, in the case of magainin, it was found that increasing the net charge to +5 also increased its antimicrobial activity, but a further increase to +7 did not result in additional increases in activity, although haemolytic activity was increased [14]. The uncharged Thr^8^ and the negatively-charged Glu^4^ and Asp^12^ of frenatin 4.2, were all substituted by Leu in the case of frenatin 4.2a, resulting in increased hydrophobicity (from 0.315 to 0.599), net charge (from +3 to +5) and helicity (from 42.58% to 61.87%), although the hydrophobic moment was decreased (from 0.525 to 0.294) because of the leucine substitutions on the hydrophilic face of the peptide. Frenatin 4.2a showed greatly improved antimicrobial activities against all three test microorganisms. This broad specificity or lower selectivity in cell killing activity of frenatin 4.2a also caused higher haemolytic activity, compared to its naturally-occurring peptide parent, frenatin 4.2. Nevertheless, despite the increase in haemolytic activity, the extent of haemolysis caused by frenatin 4.2a at its respective MICs for different microorganisms was negligible. None of the other three peptides displayed observable haemolytic activity in the tested concentration range, which correlated with their weak antimicrobial activities.

Although all those factors work dependently to affect the antimicrobial activity of antimicrobial peptides, there is an interesting correlation for the frenatin peptide family shown in this study. Frenatin 4.1a and 4.2 contain the same net charges, similar hydrophobic moment and the same level of antimicrobial activity, though their hydrophobicity and helicity are different. This suggests that net charge takes more responsibility for the potency of antimicrobial activity of frenatins, compared with frenatin 4.1 and 4.2a. It is speculated that the frenatins could lose their antimicrobial activity when the net charges are less than three, and it may explain why frenatin 4 has no antimicrobial activity against various bacteria [6]. However, in frenatin 4.2a, with more net charge and significant changes of hydrophobicity, hydrophobic moment and helicity, the activity against *E. coli* and *C. albicans* showed four-fold and 16-fold increase, respectively, and a more than 32-fold increase of anti-*S. aureus* activity and severer haemolytic activity exhibited in the meantime. The results indicate that it is more essential to consider the sites of engineered amino acid residue rather than the total number of net charges for the selectivity and bioactive potency, which was also concluded in a recent study by Zhang et al. [15]. Due to the scale of this study, the correlation of the factors of frenatins could not be well explained, which may need further investigation in combination with more naturally-occurring frenatins.

A number of frog species produce antimicrobial peptides that have similar structure to those peptides that were discovered from other frog species [16]. However, for the peptides discovered in *L. infrafrenata* to date, only ceruletide, which simulates smooth muscle and increases digestive secretions, is identical and widely-distributed in all of the Australian green tree frogs [6]. This peculiarity that bioactive peptides from *L. infrafrenata* are quite unique led to further research in the study of their skin secretion peptides. Despite this, the data from the present study have shown that rational modifications of naturally-occurring peptides with low antimicrobial activities can result in generation of more potent analogues, which could be utilized as lead compounds for the development of new drugs.

## 4. Materials and Methods 

### 4.1. Specimen Biodata and Secretion Acquisition

Specimens of *L. infrafrenata* (*n* = 4, two males and two females, 6 cm and 8 cm snout-to-vent lengths) were obtained from a commercial source in the PeruBiotech E.I.R.L., Lima, Santiago de Surco, Peru. The frogs were kept in a vivarium at 25 °C under a 12 h/12 h day/night cycle and were fed crickets three times per week. Their skin secretions were harvested after the frogs had been maintained under these conditions for around 4 months.

The skin secretions from frogs were obtained by mild transdermal electrical stimulation as previously described [17]. The secretions produced were washed from the skins into a chilled glass beaker using distilled deionised water and were subsequently snap-frozen in liquid nitrogen and lyophilised. The lyophilised sample was stored at −20 °C. This method was adopted because it was not harmful to the frogs and produced minimal stress to the animals. All procedures were carried out under appropriate animal experimentation (UK) licenses and were subject to ethical approval.

### 4.2. “Shotgun” Cloning of Skin Secretion Peptide Precursor-Encoding cDNAs

As the 5′- untranslated region of the previously-cloned caerin cDNAs from *Litoria caerulea* [9] and of aurein from *Litoria aurea* [18] were found to be highly-conserved, a primer, Litoria-S1 (5′-GVCTTGTAAAGACCAAVCATG-3′), which was previously-designed according to this region within our group [18], was adopted as a sense primer.

Lyophilised skin secretion (5 mg) was dissolved in 1 mL of cell lysis/binding buffer from a Dynabeads^®^ mRNA DIRECT™ Kit (Dynal Biotech, Merseyside, UK). The mRNAs were thus released and stabilized. Polyadenylated mRNA was trapped and isolated using magnetic oligo-dT beads and reverse transcribed to obtain a cDNA library. By using a SMART-RACE kit (Clontech, Palo Alto, CA, USA) and employing the NUP (5′-AAGCAGTGGTATCAACGCAGAGT-3′) that was supplied with the kit and the designed Litoria-S1 as a pair of primers to perform the 3′-RACE procedure, full-length peptide precursor nucleic acid sequence data was obtained. The RACE products were purified using an E.Z.N.A.^®^ Cycle-Pure Kit (Omega Bio-Tek, Norcross, GA, USA), cloned into a pGEM-T vector (Promega Corporation, Southampton, UK) and sequenced using an ABI 3100 automated capillary sequencer (Applied Biosystems, Foster City, CA, USA)

### 4.3. Identification and Structural Analysis of Mature Peptides in Skin Secretion

A further 10 mg of lyophilised skin secretion were dissolved in 1.5 mL of 0.05/99.95 (*v*/*v*) trifluoroacetic acid (TFA) /water and then clarified by centrifugation. The supernatant (1 mL) was then subjected to reversed phase HPLC using a Waters gradient reversed phase HPLC system, fitted with an analytical column (Jupiter, C_5_, 300 Å, 5 μm, 4.6 mm × 250 mm, Phenomenex, Macclesfield, Cheshire, UK). Column elution was achieved with a gradient formed from 0.05/99.95 (*v*/*v*) TFA/water to 0.05/29.95/70.00 (*v*/*v*/*v*) TFA/water/acetonitrile in 240 min at a flow rate of 1 mL/min, and the effluent was monitored by UV absorbance at 214 nm 280 nm. The eluted fractions were collected at 1 min intervals. The molecular masses of peptides in each fraction were further analysed by use of a MALDI-TOF mass spectrometer (Voyager DE, PerSeptive Biosystems, Foster City, CA, USA) in positive detection mode using α-Cyano-4-hydroxycinnamic Acid (CHCA) as the matrix. Fractions with peptide molecular masses coincident with those of the mature peptides predicted from the cloned cDNA, were then infused into an LCQ Fleet ion-trap electrospray mass spectrometer (Thermo Fisher Scientific, San Francisco, CA, USA) followed by trapping of appropriate ions for MS/MS fragmentation.

### 4.4. Solid-Phase Peptide Synthesis

Following the confirmation of primary structures of the cloned cDNA-encoded peptides, the wild-type peptides and their site-substituted analogues were chemically-synthesised by solid-phase Fmoc chemistry using a Tribute™ automated solid phase peptide synthesizer (Protein Technologies, Tucson, AZ, USA). After cleavage from the synthesis resin and side-chain deprotection, the peptides were purified by reversed phase HPLC and both molecular masses and MS/MS fragmentation profiles were employed to confirm the purity and authenticity of their structures.

### 4.5. Determination of Peptide Secondary Structures Using Circular Dichroism (CD) Analysis 

A JASCO J-815 CD spectrometer (Jasco, Essex, UK) was employed to perform the analysis of peptide secondary structures. Each peptide was dissolved in 10 mM ammonium acetate and 10 mM ammonium acetate with 50% TFE, respectively, to reach a concentration of 100 μM, in a 1 mm high precision quartz cell (Hellma Analytics, Essex, UK). All CD spectra were obtained at 20 °C from 250 nm to 190 nm at a scanning speed of 100 nm/min. The bandwidth was 1 nm, and the data pitch was 0.5 nm. K2D3 webserver [19] was used to estimate the α-helix content of the peptides from their CD spectrum data.

### 4.6. Prediction of Peptide Physicochemical Properties

Online bioinformatic tools were used to predict the physicochemical parameters of the peptides. HeliQuest CompuParam version 2 [20] was the main programme used. Helical Wheel Projections [21] could more readily visually display the amphipathic nature of peptides and the hydrophobicity of amino acid residues.

### 4.7. Minimal Inhibitory Concentration Assays

Antimicrobial activity of the peptides was monitored by determination of minimal inhibitory concentrations (MICs) using standard model microorganisms: the Gram-positive bacterium, *Staphylococcus aureus* (NCTC 10788), the Gram-negative bacterium, *Escherichia coli* (NCTC 10418) and the yeast, *Candida albicans* (NCPF 1467). The peptides were initially dissolved in physiological PBS to yield stock solutions of 5.12 × 10^4^ μg/mL. MICs were determined within the peptide concentration range of 1 μg/mL to 512 μg/mL, obtained through dilution of the stock solutions in PBS. The assays were carried in 96-well microtiter plates and the respective concentrations of peptides and controls were inoculated with microorganism cultures (5 × 10^5^ CFU/mL). The plates were incubated for 18 h at 37 °C in a humidified atmosphere. Following this, the growth of the microorganisms was determined by measuring the optical density of the culture at 550 nm using a microplate reader (EL808, Biolise BioTek, Winooski, VT, USA). MICs were defined as the lowest concentration at which no growth was detectable.

### 4.8. Haemolysis Assay

A 4% suspension of red blood cells was prepared from defibrinated horse blood (TCS Biosciences, Botolph Claydon, Buckingham, UK). The peptides were tested within the concentration range of 1 μg/mL to 512 μg/mL at 37 °C for 2 h. PBS solution was used as a negative control and an equal volume of PBS solution containing 2.0% of the non-ionic detergent, Triton X-100™ (Sigma-Aldrich) was employed as a positive control. Lysis of red cells was assessed by measuring the optical density of the sample at 550 nm using a microplate reader. The % haemolysis was calculated using the following formula:

Haemolysis% = (A − A_N_)/(A_P_ − A_N_) × 100% (1)

where A was the absorbance of the peptide sample solution and A_N_ and A_P_ were the absorbance values of the negative control and the positive control, respectively.

## Figures and Tables

**Table 1:** Physicochemical properties of frenatin peptides and their analogues.

Peptide	H	μH	Net Charge (z)	Helicity (%)
Frenatin 4.1	0.300	0.491	2	43.87%
Frenatin 4.1a	0.244	0.540	3	55.41%
Frenatin 4.2	0.315	0.525	3	42.58%
Frenatin 4.2a	0.599	0.294	5	61.87%

H represents hydrophobicity and μH represents hydrophobic moment.

**Table 2:** Minimal inhibitory concentrations (MICs) of the frenatin peptides and their analogues as determined for three different test microorganisms. Mass concentration (μg/mL) was employed, and molarity (μM) was calculated and showed in brackets.

Peptide	Minimal inhibitory concontrations (MICs)-μg/mL(μM)		
	*S. aureus*	*E. coli*	*C. albicans*
Frenatin 4.1	>512 (>202.4)	>512 (>202.4)	>512 (>202.4)
Frenatin 4.1a	>512 (>202.9)	128 (50.7)	256 (101.5)
Frenatin 4.2	>512 (>216.0)	128 (54.0)	256 (108.0)
Frenatin 4.2a	16 (6.8)	32 (13.5)	16 (6.8)

**Table 3:** Known frenatins, their sequences and sources.

Name	Sequence	Source	Reference
Frenatin 1	GLLDALSGILGL.NH_2_	*Litoria infrafrenata*	[6]
Frenatin 1.1	GLLDTLGGILGL.NH_2_	*Litoria infrafrenata*	[4]
Frenatin 2	GLLGTLGNLLNGLGL.NH_2_	*Litoria infrafrenata*	[6]
Frenatin 2D	DLLGTLGNLPLPFI.NH_2_	*Discoglossus sardus*	[7]
Frenatin 2.1D	GTLGNLPAPFPG	*Discoglossus sardus*	[7]
Frenatin 2.1S	GLVGTLLGHIGKAILG.NH_2_	*Sphaenorhynchus lacteus*	[8]
Frenatin 2.2S	GLVGTLLGHIGKAILS.NH_2_	*Sphaenorhynchus lacteus*	[8]
Frenatin 2.3S	GLVGTLLGHIGKAILG	*Sphaenorhynchus lacteus*	[8]
Frenatin 3	GLMSVLGHAVGNVLGGLFKPKS	*Litoria infrafrenata*	[6]
Frenatin 3.1	GLMSILGKVAGNVLGGLFKPKENVQKM	*Litoria infrafrenata*	[4]
Frenatin 4	GFLDKLKKGASDFANALVNSIKGT	*Litoria infrafrenata*	[6]
Frenatin 4.1	GFLEKLKTGAKDFASAFVNSIKGT	*Litoria infrafrenata*	[4]
Frenatin 4.2	GFLEKLKTGAKDFASAFVNSIK.NH*_2_*	*Litoria infrafrenata*	

## Abbreviations

The following abbreviations were used in this manuscript:

MIC	Minimal inhibitory concentration
RACE	Rapid Amplification of cDNA Ends
MALDI-TOF	Matrix Assisted Laser Desorption Ionization—Time of Flight
Fmoc	9-Fluorenylmethyloxycarbonyl
NUP	Nested Universal Primer
TFA	Trifluoroacetic Acid
CHCA	α-Cyano-4-hydroxycinnamic Acid
CFU	Colony Forming Unit
